# The Most Important Factor in Infant Mortality

**Published:** 1907-09

**Authors:** 


### THE MOST IMPORTANT FACTOR IN INFANT MORTALITY.

We have reached the season when infant mortality is the most serious with which the physician has to deal. We hear much of milk and its dangers, of crowded tenements and long-continued heat, of uncleanly quarters and garbage accumulation, but there is one factor in modern infant mortality of which we shall hear comparatively little, and yet which is of greater importance than any or perhaps even than all of these. In spite of this it is the element whose effect on the problem can be more directly traced than any other. It is the one of all the factors which, instead of being on the decrease in influence, as are practically all of the others, owing to the ameliorating hygienic effect of modern science and the spread of modern methods of sanitation, is decidedly on the increase, and threatens in the near future to be, if possible, even more pernicious than it is at the present time. This is the failure of mothers to nurse their children. Some women, it must be at once admitted, are not physically capable of supplying sufficient nourishment of the proper kind to the infant, or because the drain on their own nutritional supply would be too great for their health. It is freely conceded, however, that most women can nurse their children, but do not.

There is no substitute for mother's milk. No modification of cow's milk, no matter how carefully or scientifically made, even approaches mother's milk for the healthy nutrition of infants. This is not alone true in general, but the truth becomes even more striking if special phases of the problem are considered. No two women give milk that is absolutely the same any more than their children are alike. The milk provided by Nature for each infant is, as a general rule, of the precise composition that is best suited for the particular nature of the child. This is not an absolute rule, but the exceptions to it only demonstrate that the rule surely exists. In the face of this, the statistics with regard to nursing mothers are little short of appalling. Censuses taken in Berlin every five years contain the report of the number of mothers who have been able to nurse their own children. Of every thousand children born during the five years before 1890, 529 were nursed by their mothers. In the course of five years this number has decreased considerably, so that in 1895 only 446 children were maternally nursed. In 1900 there was a still further reduction and unfortunately in even greater proportion than for the preceeding five years. Out of over five hundred there had been a reduction of 85 for a half decade ending in 1895, but out of 446 there had been a reduction of 114 in the five years before 1900. This is practically 25 per cent., while the ratio of reduction for the preceding five years had been only a little more than 16 per cent. The figures for 1905 are said to show an even greater decrease for the first five years of the twentieth century. This would mean that less than one among four mothers in Berlin, an average of less than one, now nurses her children.

German women are said to be more willing, as a rule, to nurse their children than those of most other nationalities. Of course, city women are much more ready to avoid this duty than are those who live in the country, but it is more than probable that the state of affairs found in Berlin is not worse than that which is developing at the present moment in our own large cities in this country. It is evident that, if the high death rate among so-called nursing children, that is, those who should be nursed, is to be reduced, the influence of this factor must be guarded against much more than it has been in the past. Physicians are probably more ready to find excuses for women for not nursing their children than was formerly the case, and this has had a wide-spread influence in strengthening the present fashion which proclaims it quite the thing for a woman not to nurse her own child. There would seem to be in this a serious duty of instruction as to maternal obligations, which the medical profession must take to heart, if the present unnatural tendency to give the infant over to the dangers of artificial feeding is not to be allowed to work even more harm in the future than it has in the past.—Ed. Jour. A. M. A., July 20th, 1907.

